# Circulating maternal chimeric cells have an impact on the outcome of biliary atresia

**DOI:** 10.3389/fped.2022.1007927

**Published:** 2022-09-20

**Authors:** Ryuta Masuya, Toshihiro Muraji, Sami B. Kanaan, Toshio Harumatsu, Mitsuru Muto, Miki Toma, Toshihiro Yanai, Anne M. Stevens, J. Lee Nelson, Kazuhiko Nakame, Atsushi Nanashima, Satoshi Ieiri

**Affiliations:** ^1^Division of the Gastrointestinal, Endocrine and Pediatric Surgery, Department of Surgery, Faculty of Medicine, University of Miyazaki, Miyazaki, Japan; ^2^Department of Pediatric Surgery, Research Field in Medicine and Health Sciences, Medical and Dental Sciences Area, Research and Education Assembly, Kagoshima University, Kagoshima, Japan; ^3^Research and Development, Chimerocyte, Inc., Seattle, WA, United States; ^4^Clinical Research Division, Fred Hutchinson Cancer Research Center, Seattle, WA, United States; ^5^Department of Pediatric Surgery, Ibaraki Children's Hospital, Mito, Japan; ^6^Seattle Children's Research Institute, University of Washington, Seattle, WA, United States; ^7^Division of Hepato-Biliary-Pancreatic Surgery, Department of Surgery, Faculty of Medicine, University of Miyazaki, Miyazaki, Japan

**Keywords:** biliary atresia, maternal microchimerism, qPCR, prognosis, GVHD, autoimmunity, Treg

## Abstract

**Introduction:**

We aimed to quantify the DNA of maternal chimeric (MC) cells in the peripheral blood of the BA patients and investigated the impact on the outcome.

**Methods:**

Patients with progressive jaundice because of no bile flow, which necessitated liver transplantation, or who showed inadequate bile flow with or without episodes of cholangitis and progressive hepatic fibrosis and portal hypertension were classified into the poor group. Those with adequate bile flow with completely normal liver function tests beyond 2 years were classified into the good group. The qPCR were separately carried out in buffy coat samples and plasma samples, targeting the non-inherited maternal HLA alleles in the DNA samples.

**Results:**

MC-DNA was present in the buffy coat (10–328 gEq per 10^6^ host cells) in seven patients. There was no MC-DNA in the remaining five patients. MC-DNA (214–15,331 gEq per 10^6^ host cells) was observed in the plasma of five patients. The quantity of MC-DNA in the buffy coat showed a significant difference between the two prognostic groups (*p* = 0.018), whereas there was no significant difference in the quantity of MC-DNA in plasma (*p* = 0.205). MC-DNA in the buffy coat was significantly associated with the outcome (*p* = 0.028), whereas MC-DNA in the plasma did not influence the outcome (*p* = 0.56).

**Conclusions:**

Poor outcomes in BA were correlated with circulating maternal chimeric lymphocytes.

## Introduction

The etiology of biliary atresia (BA) is unknown. Based on maternal microchimerism (MMc) detected in the liver of the patients (1–4), materno-fetal immune interaction has been proposed for the pathogenesis of BA. However, whether maternal chimeric (MC) cells circulate in the peripheral blood and the roles of MC cells in the etiopathogenesis of BA remains to be clarified. We aimed to quantify MC-DNA in the peripheral blood and investigated the impact on the outcome of BA patients.

## Methods

The study population included 12 patients with BA. All patients received Kasai portoenterostomy (KPE) more than 2 years before this analysis. Patients with progressive jaundice because of no bile flow, which necessitated liver transplantation, or who showed inadequate bile flow with or without episodes of cholangitis and progressive hepatic fibrosis and portal hypertension were classified into the poor group. Those with adequate bile flow with completely normal liver function tests beyond 2 years were classified into the good group.

### HLA genotype analysis

In order to define non-inherited maternal antigens (NIMA) used for marker of chimeric maternal DNA in each patient, HLA genotypes of the patients and their mothers were determined at HLA Foundation Laboratory (2F #1 Kyoto Research Park, Shimogyo-ku, Kyoto, Japan), using peripheral blood or buccal mucosa samples.

### Quantitative PCR-based high resolution chimerism assays

Mononuclear cells (buffy coat) and plasma were isolated by density gradient centrifugation (800 g, for 20 min at room temperature) of fresh heparinized whole blood using Lymphoprep™. We extracted DNA from each sample using QIAmp®. Samples from the buffy coat and plasma were separately stored in frozen and sent to Fred Hutchinson Cancer Research Center (FHCRC) and Chimerocyte, Inc. Seattle, Washington, USA, without providing clinical information of each patient. Quantitative PCR primers of non-inherited maternal antigens are available (HLA B^*^13, B^*^44, DRB1^*^01, DRB1^*^04, DRB1^*^08, DRB1^*^09, DRB1^*^14, and DRB1^*^15/^*^16) in FHCRC. The real-time qPCR reactions were separately carried out in buffy coat samples and plasma samples, targeting the non-inherited maternal HLA alleles in the DNA samples for a chimerism detection assay from a panel of qPCR-based highly sensitive and polymorphism-specific methods. We divided each sample into six wells, measured each of them, and then determined the average value. Maternal microchimerism was expressed as the genomic equivalent in 10^6^ gEq of host DNA. The gEq corresponds to 6.6 pg of human genomic DNA (5).

### Statistical analyses

Gender composition by prognostic group and differences in outcome with and without MMc were analyzed with the 2 × 2 Fisher Exact test, respectively. Current age and age in days at KPE were compared between prognostic groups using *t*-tests. Age at sampling and DNA content were compared between prognostic groups using the Mann-Whitney *U*-test. *P*-values of <0.05 were considered statistically significant.

This study was performed after receiving institutional review board approval from the local ethics committee of Kagoshima University (Registration Number: 170248) and Ibaraki Children's Hospital (Registration Number: 28A-4).

## Results

**Figure 1:**
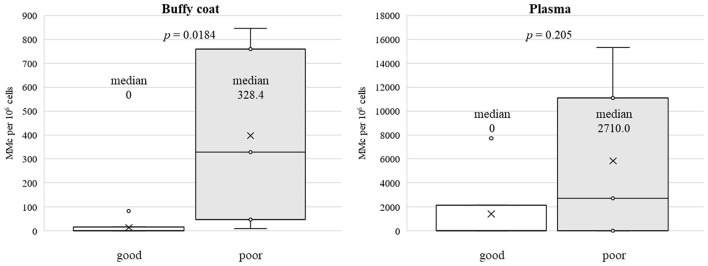
A significant difference was observed between the two prognostic group in the quantity of MC-DNA in the buffy coat (median, 0 vs. 328.4, *p* = 0.018), whereas the quantity of MC-DNA in the plasma did not differ to a statistically significant extent (median, 0 vs. 2710.0, *p* = 0.205).

Demographics of patients and quantified MC-DNA are summarized in Table 1. There were no significant differences in patient background between the two groups. Gender composition was two males and five females in the good group and two males and three females in the poor group (*p* = 1). The current age averaged 7.1 ± 3.1 years in the good group and 7.6 ± 6.1 years in the poor group (*p* = 0.87). Age at KPE averaged 67.4 ± 20.3 days in the good group and 59.2 ± 9.0 days in the poor group (*p* = 0.42). Age at sampling was a median of 4 years (98 days−11 years) for the good group and 4 years (73 days−14 years) for the poor group (*p* = 1). MC-DNA was present in the buffy coat (10–328 gEq per 10^6^ host cells) in seven patients. There was no MC-DNA in the remaining five patients. MC-DNA (214–15,331 gEq per 10^6^ host cells) was observed in the plasma of five patients. The quantity of MC-DNA in the buffy coat showed a significant difference between the two prognostic groups (*p* = 0.018), whereas there was no significant difference in the quantity of MC-DNA in plasma (*p* = 0.205; Figure 1). MC-DNA in the buffy coat was significantly associated with the outcome (*p* = 0.028), whereas MC-DNA in the plasma did not influence the outcome (*p* = 0.56).

**Table 1:** Demographics, quantities of maternal chimeric DNA, and the prognosis of each patient.

**#**	**Sex**	**Age**	**Days at KPE**	**Age at sampling**	**Buffy coat**			**Plasma**			**Prognosis**	**Clinical course**
					**Total cell assayed (gEq)**	**Maternal chimeric cells (gEq/10^6^)**	**95%CI of maternal chimeric cells**	**Total cell assayed (gEq)**	**Maternal chimeric cells (gEq/10^6^)**	**95%CI of maternal chimeric cells**		
1	F	3	98	98 days	15,713.7	0.0	0.0–247.7	246.3	0.0	0–17,011.7	Good	No episodes of cholangitis
2	F	5	39	3 y/o	184,333.6	0.0	0.0–21.0	209,033.2	0.0	0–18.6	Good	No episodes of cholangitis
3	F	6	62	4 y/o	54,534.4	0.0	0.0–70.4	465.6	2,147.9	379.3–12,065.4	Good	No episodes of cholangitis
4	M	7	83	6 y/o	158,799.9	0.0	0.0–24.3	258,540.8	0.0	0–14.9	Good	No episodes of cholangitis
5	F	8	80	4 y/o	26,108.3	0.0	0.0–190.4	1,205.5	0.0	0–3,176.5	Good	Two episodes of cholangitis only during the first post-operative year
6	M	8	56	4 y/o	63,192.7	82.7	35.9–190.9	137,949.8	0.0	0–28.1	Good	No episodes of cholangitis
7	F	13	54	11 y/o	116,333.1	17	4.6–64.0	258.3	7,743.9	2,001.0–29,611.2	Good	No episodes of cholangitis
8	F	2	73	73 days	19,773.1	328.4	152.6–706.6	326.1	15,330.9	6,451.6–36,006.8	Poor	Cholangitis
9	M	3	61	5 m/o	102,403.1	9.8	1.7–55.6	1,107	2,710	913.5–8,012.3	Poor	Severe suppurative cholangitis, OLT
10	M	5	59	4 y/o	107,391.5	46.9	19.7–111.3	136,027.8	0.0	0–28.4	Poor	Cholangitis
11	F	12	54	9 y/o	2,631.8	759.9	208.4–2,766.8	179.8	11,122.6	3,055.5–39,641.7	Poor	Frequent cholangitis, with bile lake
12	F	16	49	14 y/o	2,361.4	847	224.1–3,199.7	70	0.0	0–52,164.4	Poor	Gastrointestinal bleeding, portal hypertension

We express cell count units in terms of human genome equivalents (gEq). We also describe the amount of maternal chimeric cells in gEq per 10^6^ host cells. DNA in the buffy coat reflects the number of lymphocytes and DNA in the plasma represents the number of maternal cells incorporated in the host tissue that shed their DNA.

## Discussion

It is not clearly understood whether MMc is a direct or indirect cause of autoimmune diseases or simply a bystander-associated phenomenon. MC cells in the BA liver include CD8+, CD45+, and cytokeratin-positive cells (4). A direct cause implies GVHD-like immune interaction (CD8+ effector lymphocytes are MC cells), whereas an indirect cause implies that MC cells are cytokeratin-positive cells, which are targets of autologous T lymphocytes. The other possible indirect cause is regulatory T cell (Treg) dysfunction (6–8), which is affected by MMc, leading to host immune responses toward self-antigens. To distinguish these alloimmune reactions, we quantified MC-DNA separately in the buffy coat, which reflects the number of maternal lymphocytes and in the plasma, representing the number of MC cells incorporated in host tissue that shed their DNA.

To the best of our knowledge, this is the first study to demonstrate that MC lymphocytes persist in circulation in some patients with BA and that they are associated with poor outcomes, suggesting that maternal cells are not bystanders.

The number of gEq measurements in the plasma is widely variable. In plasma, we measure circulating DNA and estimate the cell count by counting how many copies of the reference gene are appearing (in our case, the B-globin gene). This gives a reasonable estimate of how many cells have died and shed their DNA into the plasma. Many factors can affect the amount of total circulating DNA, including inflammation, the state of immune cells undergoing apoptosis, and other tissue cells dying. Therefore, the range can be broad, mainly if the cohort consists of diseased subjects.

Tamaoka et al. (9) recently conducted an MC-DNA assay of samples from the left lateral segment of the explanted liver; the analysis was identical to that developed in FHCRC. MC-DNA was not significantly more frequent in these livers, and they concluded that MMc played no significant role in the etiology of BA. As the left lateral segment of the BA liver is a unique segment associated with more severe inflammatory reactions and fibrosis (10), MMc may indirectly predispose offspring to autoimmunity, such as inhibition of the Treg function.

The limitation of this study is that samples were collected from each patient at only one-time point. Collecting samples at multiple time points and investigating changes over time would allow further clarification of the behavior of MC cells in BA patients.

## Conclusions

Poor outcomes in BA were correlated with circulating maternal chimeric lymphocytes. Future investigation is warranted to clarify the immunological dysregulation associated with MMc.

## Data availability statement

The original contributions presented in the study are included in the article/supplementary material, further inquiries can be directed to the corresponding author.

## Ethics statement

The studies involving human participants were reviewed and approved by Ethics Committee on Clinical Research, Sakuragaoka Campus, Kagoshima University Ethics Committee on Clinical Research, Ibaraki Children's Hospital. Written informed consent to participate in this study was provided by the participants' legal guardian/next of kin.

## Author contributions

RM, TM, and SI conceptualized and designed the study, drafted the initial manuscript, and reviewed and revised the manuscript. TH, MM, MT, TY, and KN collected samples and revised the manuscript. RM and SK carried out the initial analyses and revised the manuscript. AS, JN, and AN conceptualized and designed the study, coordinated and supervised data collection, and critically reviewed the manuscript for important intellectual content. All authors approved the final manuscript as submitted and agree to be accountable for all aspects of the work.

## Funding

This project was supported by a Grant-in-Aid for Exploratory Research (KAKEN C ID: 17K11514).

## Conflict of interest

Authors SK and JN are co-founders of Chimerocyte, Inc. The remaining authors declare that the research was conducted in the absence of any commercial or financial relationships that could be construed as a potential conflict of interest.

## Publisher's note

All claims expressed in this article are solely those of the authors and do not necessarily represent those of their affiliated organizations, or those of the publisher, the editors and the reviewers. Any product that may be evaluated in this article, or claim that may be made by its manufacturer, is not guaranteed or endorsed by the publisher.
